# Could FIV zoonosis responsible of the breakdown of the pathocenosis which has reduced the European CCR5-Delta32 allele frequencies?

**DOI:** 10.1186/1743-422X-5-119

**Published:** 2008-10-16

**Authors:** Eric Faure

**Affiliations:** 1 LATP, CNRS-UMR 6632, IFR48 Infectiopole, Evolution biologique et modélisation, case 5, Université de Provence, Place Victor Hugo, 13331 Marseille cedex 3, France

## Abstract

**Background:**

In Europe, the north-south downhill cline frequency of the chemokine receptor CCR5 allele with a 32-bp deletion (*CCR5-Δ32*) raises interesting questions for evolutionary biologists. We had suggested first that, in the past, the European colonizers, principally Romans, might have been instrumental of a progressively decrease of the frequencies southwards. Indeed, statistical analyses suggested strong negative correlations between the allele frequency and historical parameters including the colonization dates by Mediterranean civilisations. The gene flows from colonizers to native populations were extremely low but colonizers are responsible of the spread of several diseases suggesting that the dissemination of parasites in naive populations could have induced a breakdown rupture of the fragile pathocenosis changing the balance among diseases. The new equilibrium state has been reached through a negative selection of the null allele.

**Results:**

Most of the human diseases are zoonoses and cat might have been instrumental in the decrease of the allele frequency, because its diffusion through Europe was a gradual process, due principally to Romans; and that several cat zoonoses could be transmitted to man. The possible implication of a feline lentivirus (FIV) which does not use CCR5 as co-receptor is discussed. This virus can infect primate cells *in vitro *and induces clinical signs in macaque. Moreover, most of the historical regions with null or low frequency of *CCR5-Δ32 *allele coincide with historical range of the wild felid species which harbor species-specific FIVs.

**Conclusion:**

We proposed the hypothesis that the actual European CCR5 allelic frequencies are the result of a negative selection due to a disease spreading. A cat zoonosis, could be the most plausible hypothesis. Future studies could provide if CCR5 can play an antimicrobial role in FIV pathogenesis. Moreover, studies of ancient DNA could provide more evidences regarding the implications of zoonoses in the actual *CCR5-Δ32 *distribution.

## Background

As infection is the greatest killer in human history [1], the strongest evidence for selection in the human genome has been obtained for genes involved in immune defense, including those which encode receptors. One of the most-celebrated examples of adaptive selection is the 32-bp coding sequence deletion, *CCR5-Δ32*, of the chemokine receptor CCR5. This is probably the more recent and complete example of a gene studied from clinical, epidemiological and evolutionary genetics. CCR5 function as co-receptors for the cell entry of HIV-1 and the deletion which leads to a frame shift and generates an inactive CCR5 receptor. Homozygosity for the *CCR5-Δ32 *allele confers almost complete, mendelian resistance to R5-tropic HIV-1 while HIV-infected individuals heterozygous for this allele were delayed in progression to AIDS [2,3].

**Figure 1:**
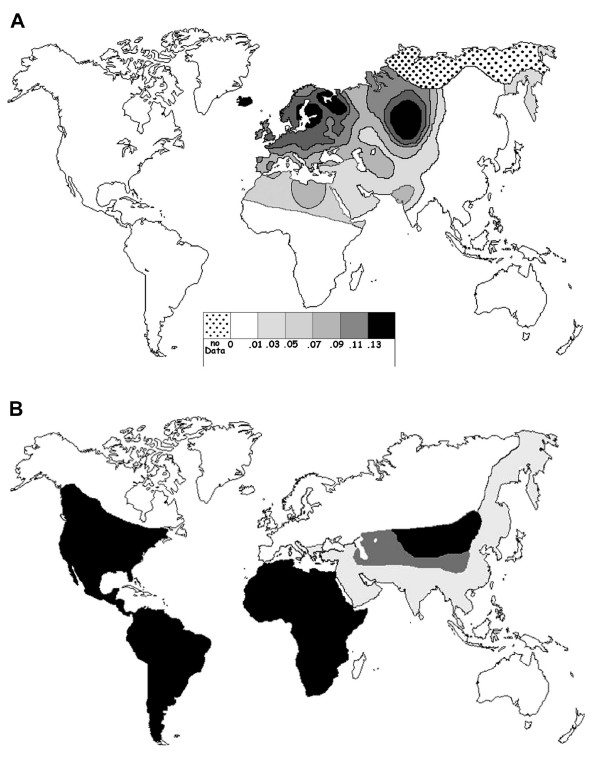
**Geographic distribution of the *CCR5-Δ32 *allele (A) compared with historical range of felids carrying species-specific FIVs (B)**. In (A), only the frequencies of Native populations have been evidenced in America, Asia, Africa and Oceania. Map redrawn and modified from [4,5]. In (B), the black areas correspond to the range of wild individuals bearing species-specific FIVs in a given continent, America: bobcat, jaguarundi, ocelot and puma; Asia: Pallas cat; Africa: cheetah, leopard and lion. The pale grey areas correspond to the range where individuals of these species have been found seronegative or when their serological status is unknown in a given continent (Asia: cheetah, leopard and lion; Europe: leopard and lion). Areas where these last three species lived in sympatry with Pallas cat are in dark grey. The historical ranges are approximate by 500 BC for Europe, North Africa and Western Asia; since the European settlement in America, and during the 1500's to the beginning of the 1900's in the remainder of Africa, Asia and Oceania. These data were principally inferred from [65-71].

The *CCR5*-*Δ32 *allele is mainly present in Europeans (10% on average) and the allele frequency exhibits a north-south cline with frequencies ranging from 16% in Northern Europe to 4% or less in Greece and in most of the Mediterranean islands (Figure 1A and [4,5]). The broadest area of high frequency is located in North-Eastern Europe, particularly in the Baltic and White Sea regions. From these maximum, the frequency gradually decreases in all directions across Europe [4]; however, some additional peaks of frequency are found in France or Russian areas [4,6-8]. Moreover, Ashkenazi Jews have high frequencies of *CCR5-Δ32*, but this is likely due to founder effects unique to their history rather than the general process of dispersal that spread the allele in other populations [9]. Outside Europe, the mutation can be found at low frequencies in neighbouring regions (North Africa, Middle East, Central Asia); it is absent in Sub-Saharan Africa, East and South-East Asia and in indigenous populations of America and Oceania (Figure 1A).

Because the AIDS pandemic is too recent to change allele frequencies, other infectious diseases have been suggested as the agent causing the selection of the null allele increase, such as resistance to plague and smallpox infections [10]. However, analyses of Scandinavian Mesolithic DNA which have pushed the date of the first occurrence back to around 5000 BC [11] and genomic analyses [12] have weakened the evidence for recent selection of the null allele. Due to the north-south spatial gradient, it has been proposed that the actual allele distribution could be explained by migrations of Northern populations. As suggested by Lucotte [13] in its seminal article in the field and by Balanovsky et al. [4] Vikings and Uralic speaking people, respectively, could have brought the deletion in some Southern populations. Moreover, these migrations and/or gene flow cannot explain, according to us, the whole of the European allele frequency distribution. Also, we have proposed an alternative hypothesis in which the actual allele frequency distribution might not be due to the genes spreading, but to a negative selection resulting in the spread of pathogens principally during principally Roman expansion [5]. This hypothesis is supported by several facts.

The idea that bottlenecks and founder effects could lead to an increase in damaging alleles in human populations was historically reserved for isolated populations that experienced severe founder effects (for example, Ashkenazi Jews [14] and Finns [15]). However, recently signs of a population bottleneck in variability data obtained for a number of genomic loci in European populations were described and also led to the conclusion that a severe bottleneck occurred after the appearance of the anatomically modern human in Africa, and thus presumably during, or after, the emigration out of Africa [16-18] and references therein). Moreover, the earlier European population of hunter-gatherers could suffer severe bottlenecks during the latest ice age (Pleistocene) [19]. As there is strong evidence for the unitary origin of the *CCR5-Δ32 *mutation [20,21], the null allele could have been already present in the ancestors of the European populations (in spite of their present language differences) at a relatively high frequency, probably >10% as suggested by analysis of ancient DNA from Bronze age [22] and Neolithic [11], similarly to many other polymorphisms found in Europeans but not in the other populations [23].

Previous statistical analyses showed strong negative correlations in Europe between the allele frequency and two historical parameters, i.e. the first colonization dates by the great ancient Mediterranean civilisations, and the distances from the frontiers of the Roman Empire in its greatest expansion [5]. However, the possible decrease of the ancestral *CCR5-Δ32 *allele frequency was not due directly to the colonizers, as the gene flows to European native populations were extremely low [19]. This suggests that the role of colonizers were indirect. As evolutionary biologists have shown several evidences that infectious diseases, as a leading cause of human morbidity and mortality, have exerted important selective forces on our genomes [24,25], the cause of the decrease of the *CCR5-Δ32 *allele frequency in Southern European populations is probably due to infectious agent(s). It has been suggested that the most important infectious diseases of modern food-producing human populations also include diseases that could have emerged only within the past 11,000 years, following the rise of agriculture [1,25,26]. The second great historical transitions occurred when great ancient conquering Eurasian civilizations (such as the Greek and Roman empires) came into military and commercial contact, *ca*. 3000–2000 years ago, swapping their dominant infections [27]. It is either a human disease or a zoonosis transmittable to humans. Moreover, studies on the West Nile virus have shown that host genetic factors are highly pathogen-specific and can therefore be beneficial in one context and harmful in another [28]. Which agree that the possible decrease of the *CCR5-Δ32 *allele frequency in the South of the Europe could be due to parasites. The introduction of parasites in naive colonized populations could have induced a breakdown of the pathocenosis and a new equilibrium has been reached through a decrease of the *CCR5-Δ32 *allele frequency. The theoretical framework of pathocenosis, first coined by Grmek [29,30]) and developed by Biraben [31], offers a synthetic approach to the history of disease. Drawing on the concept of biocenosis, Grmek defines pathocenosis as "the ensemble of pathological states present in a specific population at a given moment in time" and suggests that "the frequency and overall distribution of each disease depends on the frequency and distribution of all the other diseases within a given population". The concept of pathocenosis attempts to offer a synthetic view of disease ecology, which, in our context is defined as all interdependences within pathogens, their hosts (including their genetic responses) and their environment.

The aim of this article is to critically discuss the possible nature of this (or these) parasite(s) responsible of the decrease of the *CCR5-Δ32 *allele frequency in the Southern European populations.

## Results

### Putative role of cats as host-parasite

Previous, statistical analyses suggested a decrease of the ancestral *CCR5-Δ32 *allele frequency in European population due principally to Roman expansion [5]. However, this negative selection was not directly due to the military or colonisation spreads, as the gene flows from colonisators to European native populations were too low [19]. Moreover, statistical analyses suggested that factor(s) responsible for the decrease of null allele frequency had partly diffused beyond the borders of the Roman Empire [5]. The diffusion of one or more factor(s) excludes the role of climatical changes, the change in allele frequency could be due to the spread of human or animal parasites that affect human populations.

More than any other civilizations, the Romans have created links between Mediterranean basin and Western and Central Europe and the great routes of infectious diseases went straight through it [32]. Not only did the first great historical pestilences pass through the Empire, but also the slow insidious penetration of endemic disease (like tuberculosis, leprosy and malaria) has invaded Europe [30]. Moreover, conquerors and invading armies brought also with them insect and rodent vectors that could introduce or sustain infectious diseases in nonendemic European areas. As, to our knowledge, no known human diseases could explain the decrease of the null allele in Europe, zoonoses might be implicated. Indeed, most of the infectious diseases affecting human populations are considered zoonotic in origin [33-35]. Close contact with animals is a risk for humans to acquire infectious diseases and it is well known that the domestication of animals has facilitated the passage of animals parasites to human [36,37]. Many of the major human infectious diseases, including some now confined to humans and absent from animals, have arisen only after the origins of agriculture (11,000 years BP) [1,25,26]. The five animal species (cow, sheep, goat, pig and dog) which have had probably the most epidemic impact on the human populations are explicitly named the Pandora's pentad [38]. Moreover, few tropical but many temperate diseases arose from domestic animals, because these live mainly in the temperate zones, and their concentration there was formerly even more lopsided [35]. In Europe, the Romans were the cause of some permanent changes in the distribution of birds and beasts [39]; several animals, such as cat, donkey, mule and pheasant have been voluntarily introduced throughout Europe [40] and others involuntarily, such as malaria vector mosquito species [30]. If we consider that the most impact on the decrease of the *CCR5-Δ32 *allele frequency could be principally due to Roman expansion, according to us, among all the domestic animals, cat could be the best candidate. Indeed, once the cat had arrived in Rome; this animal would have spread throughout Europe, quite likely as a camp follower and companion to the constantly travelling Roman armies. Moreover, several parasitic, bacterial and viral zoonoses diagnosed in cats could be transmitted to man [41,42]. To support this view, before investigate the type of disease which could be transmitted, the major steps of the spread of the domestic cat in Europe are summarized.

Cat's origin is yet little uncertain; however, several analysis revealed that cats were domesticated in the Near East; wildcats of Near East (*F. s. lybica*) are the closest group to all domestic cats [43,44], and likely coincide with agricultural village development in the Fertile Crescent. This is congruent with archaeological studies, the earliest evidence of cat-human association involves their co-occurrence in Cyprus deposits aged at 9,500 years ago [45]. Similarly, in all the other islands of the Mediterranean Basin far beyond continent (Sardinia, Corsica and Crete), felids originated from African or Middle East wildcats which were voluntarily introduced by Neolithic navigators about 6,000–8,000 years ago [46-50]. Interestingly, the populations of these areas have the lower level of *CCR5-Δ32 *allele frequency (references therein [5]. The earliest records of probably tamed or domestic cats in continental Europe would be in Greece by 1000 BC; however, at that time, cats were very extremely rare until 6^th^–5^th ^centuries BC [51-53]. In the Italian Peninsula, first historical evidence of tamed or early-domesticated cats was found on archaeological sites from the beginning of the 5^th^–4^th ^centuries BC [50,54]. Interestingly, in numerous parts of the Roman Empire, generally the oldest remains of the domestic cat (for example in Belgium, Netherlands, Hungary and Switzerland) dated to the Roman period [55-59]; moreover, remains of cats have been found in many of the Roman settlements excavated extensively suggesting that the spread of domesticated cats throughout continental Europe and Great Britain is principally due to Romans [40,60]. Moreover, as contrarily to Asia, Africa and America, there was no tameable felid in the Northern Mediterranean countries, therefore numerous substitutes have been found by the European populations, principally Mustelidae species [48,56,61,62].

### World repartition of FIV-infected felids and their relationship with humans

If we hypothesize that a cat zoonosis might be transmitted to human, the corresponding infectious agent could also affect other felid species. Among, all the cat zoonoses, according us, only one parasite distribution could be correlated to those of the *CCR5-Δ32 *allele frequency. The corresponding infectious agent is the *Feline immunodeficiency virus *(FIV) which can also infect primate cells *in vitro *and induce clinical signs in a primate [63,64] and references therein). Indeed, historical regions with null or low frequency of *CCR5-Δ32 *allele coincide with historical range of the wild felid species [65-71] which harbor species-specific FIVs (Figure 1). The two maps do not correspond perfectly, and we can only conclude that these patterns are not inconsistent with the hypothesis that allele frequency and the old presence FIV-infected felids are causally related. However, as developed below, bibliographical analyses provide several arguments in favour of this hypothesis.

FIV, as *Human immunodeficiency virus *(HIV) and *Simian immunodeficiency virus *(SIV) belong to the *Lentivirus *genus of the *Retroviridae *(reviewed in [72]). In domestic cat, FIV infection results in disease progression and outcome similar to that of HIV in humans, and offers a natural model to AIDS [73,74]. Other felid species which are infected with FIV seem not to develop AIDS-like disease [75,76]. However, both captive and/or wild FIV-infected lions (*Panthera leo*) and pumas (*Puma concolor*) exhibited mild to severe CD4+ T-cell depletion and some other clinical health consequences [77-80]. These findings raise the prospect that FIV is not completely benign in these species, but rather suppress host immune response and may increase the incidence of opportunistic infections or even spontaneous cancers as AIDS does in humans.

The extant felids have arisen from a common ancestor in Asia 10.8 MYA during the Miocene. The 37 felid species form eight distinct evolutionary lineages that have successfully inhabited all continents except Oceania and Antarctica through a series of migrations likely facilitated by sea-level oscillations [81]. Among the Felidae, at least 11 free ranging Felidae species harbor FIV antibodies and FIV viral genomes (Table 1). Moreover, nine of these species (lion, cheetah, leopard, Pallas cat, jaguarundi, ocelot, domestic cat, puma, and bobcat) have been shown to harbor species-specific FIVs by evaluation of complete or partial viral genomic sequences (Table 1 and [74,82,83]). However, the seroprevalence of FIVs varies dramatically by species and geographic areas. African lion and leopard, puma and Pallas cat populations demonstrate very high rates of seropositivity. The seroprevalence of FIV infections in natural settings is nearly 100% in Serengeti African lions and pumas of Wyoming and Montana, respectively [84-86]. In contrast, significant numbers of free-ranging lions in Namibia or from Asia were all seronegative [86,87]. The absence of FIV-Ple in Namibia is puzzling, but may be explained by the low density of lions in this African area [88]. Moreover, several Asian lions held in captivity were noted to be 75% FIV seropositive, demonstrating that lions of Asian origin are not intrinsically resistant to infection [89]. Interestingly, a similar geographic dispersal of seropositivity was noted for Asian versus African leopards; i.e., free-ranging African populations demonstrate seropositivity of >25%, whereas Asian-born animals are seronegative [90,91]. More than 50% of Pallas cats (Manul) tested harboured anti-FIV antibodies [91]. Other species, including the domestic cat, cheetah, and South American Neotropical free-ranging felid populations, tend to demonstrate seroprevalence rates of 10% or less. Asian species other than the Pallas cat are apparently not infected with an endemic FIV, although when during captivity, Asian felid individuals are exposed to other species harbouring FIVs, these animals may become infected (Table 1 and [74,91]). It must be noted that a species-specific FIV-related virus has also been found in Hyaenidae, which belong to the Feloidea superfamily [91,92].

**Table 1:** List of actual felids and hyanids and their FIV status

Feloidea: Felid lineages and Hyaenidae	Species	Animal	Distribution (formerly widespread)	FIV status (Western)	FIV status (PCR)	First-known taming dates
Wildcat	*Felis silvestris silvestris *(Schreber 1777)	European wildcat	Europe, S.W. Asia	+ fr	+	N.D.
	*F. s. lybica *(Forster 1780)	Northern African wildcat	Africa, Middle East	+ fr	N.D.	<2000 B.C.
	*F. s. ornata *(Gray 1830)	Asian wildcat	W. and C. Asia	+ fr	-	<2000 B.C.
	*F. bieti *(Milne-Edwards 1892)	Chinese steppe cat	China	N.D.	N.D.	N.D.
	*F. chaus *(Schreber 1777)	Jungle cat	S. and S.E. Asia, Middle East, Egypt	+/- wb, cb	-	<2000 B.C.
	*F. margarita *(Loche 1858)	Sand cat	Africa, Arabia, S.W. Asia	+ fr	-	N.D.
	*F. nigripes *(Burchell 1824)	Black-footed cat	Africa	+/- cb	-	N.D.
						
Leopard cat	*Prionailurus bengalensis *(Kerr 1792)	Leopard cat	E. and S.E. Asia, India	+ wb	+	N.D.
	*P. planiceps *(Vigors and Horsfield 1827)	Flat-headed cat	Malatya, Sumatra, Borneo	+ fr	N.D.	N.D.
	*P. rubiginosus *(I. G S-H 1831)	Rusty-spotted cat	India, Sri Lanka	- wb	N.D.	N.D.
	*P. viverrinus *(Bennett 1833)	Fishing cat	S.E. Asia, N.E. India	+ cb	-	N.D.
	***Otocolobus manul ***(Pallas 1776)	**Pallas' cat**	C. and W. Asia	+ e, fr	+	<1000 A.D.
						
Puma	***Puma concolor ***(Linnaeus 1771)	**Puma**	N. and S. America	+ e, fr	+ fr	<1500 A.D.
	***Herpailurus yagouaroundi ***(E. G S-H 1803)	**Jaguarundi**	Mexico, C. and S. America	+ fr	+ fr	<1000 A.D.
	***Acinonyx jubatus ***(Schreber 1775)	**Cheetah**	Africa, Asia Minor, India, W. Asia	+ e, fr	+ fr	<2000 B.C.
						
Lynx	*Lynx canadensis *Kerr 1792	Canada lynx	N. America	- fr	N.D.	N.D.
	*L. lynx *(Linnaeus 1758)	Eurasian lynx	Europe and Asia	- wb	N.D.	N.D.
	*L. pardinus *(Temminck 1827)	Iberian lynx	Spain and Portugal	- fr	-	N.D.
	***L. rufus ***(Schreber 1777)	**Bobcat**	N. America	+ e, fr	+	N.D.
						
Ocelot	***Leopardus pardalis ***(Linnaeus 1758)	**Ocelot**	C. and S. America, Mexico	+ fr	+	<1500 A.D.
	*L. colocolo *(Molina 1782)	Pampas cat	S. America	+ fr	-	N.D.
	*L. geoffroyi *(d'Orbigny and Gervais 1844)	Geoffroy's cat	S. America	+ e, fr	-	<1500 A.D.
	*L. guigna *(Molina 1782)	Kodkod	C. Chile, Andean Argentina	- cb	N.D.	N.D.
	*L. jacobita *(Cornalia 1865)	Andean mountain cat	Parts of Andes	N.D.	N.D.	N.D.
	*L. tigrinus *(Schreber 1775)	Tigrina	S. America	+ e, fr	-	N.D.
	*L. wiedii *(Schinz 1821)	Margay	C. and S. America	+ e, fr	+	<1500 A.D.
						
Caracal	*Caracal caracal *(Schreber 1776)	Caracal	Africa, Middle East, S.W. Asia	- wb, cb	N.D.	<1500 A.D.
	*C. aurata *(Temminck 1827)	African golden cat	Africa	+/- wb, cb	-	N.D.
	*Leptailurus serval *(Schreber 1776)	Serval	Africa	- fr	N.D.	<1500 A.D.
						
Bay cat	*Catopuma badia *(Gray 1874)	Bornean bay cat	Borneo	- cb	N.D.	N.D.
	*C. temminckii *(Vigors and Horsfield 1827)	Asian golden cat	Asia	+/- wb, cb	-	N.D.
	*Pardofelis marmorata *(Martin 1837)	Marbled cat	S.E. Asia	+/- wb, cb	-	N.D.
						
Panthera	***Panthera leo ***(Linnaeus 1758)	**Lion**	Africa	+ e, fr	+	<2000 B.C.
	*P. leo *(Linnaeus 1758)	Lion	S.W. Asia	+ cb	+	<2000 B.C.
	*P. onca *(Linnaeus 1758)	Jaguar	Mexico, C. and S. America	+ e, fr	N.D.	N.D.
	***P. pardus ***(Linnaeus 1758)	**Leopard**	Africa	+ fr	+	<2000 B.C.
	*P. pardus *(Linnaeus 1758)	Leopard	Asia	+ cb	N.D.	<2000 B.C.
	*P. tigris *(Linnaeus 1758)	Tiger	India, E. and S.E. Asia	+ cb	+	~200 B.C.
	*P. uncia *(Schreber 1758)	Snow leopard	C. Asia	+ wb	+	N.D.
	*Neofelis nebulosa *(Griffith 1821)	Mainland clouded leopard	S.E. Asia	+ cb	-	N.D.
	*N. diardi *(G. Cuvier 1823)	Sunda Island clouded leopard	Sumatra and Borneo	N.D.	N.D.	N.D.
						
*Hyaeninae*	*Crocuta crocuta *(Erxleben 1777)	Spotted hyena	Africa, S. of the Sahara	+ e, fr	+ fr	N.D.
	*Hyaena Hyaena *(Linnaeus 1758)	Striped hyena	Africa but S. Africa, S.W. Asia	+ e, fr	-	<2000 B.C.
	*H. brunnea *(Thunberg 1820)	Brown hyena	S. Africa	-	N.D.	N.D.
						
*Protelinae*	*Proteles cristatus *(Sparrman 1783)	aardwolf	S. and E. Africa	N.D.	N.D.	N.D.

The data concerning taming dates and FIV status were inferred principally from the following references: [40,45,52,65,68,105-116] and [74,82,91,98,117] and references therein. Felid lineages are from Johnson et al. (2006) [81]. The names of the two sub-families of the Hyaenidae are in italic. In bold letters, species with their specific FIV strains. Abbreviations: concerning species, G S-H, Geoffroy Saint-Hilaire; concerning the distribution, C., central; E., East; N., North; S., South; concerning the FIV status, +, positive; -, negative; +/-, indeterminate; cb, captive-born (generally zoo animals); e, endemic; fr, free ranging; N.D., not done; wb, wild-born zoo animal.

**Figure 2:**
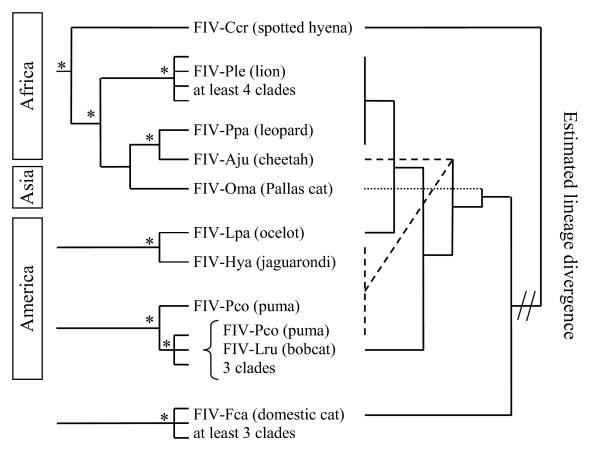
**Viral-host co-evolution**. The tree on the left shows observed viral sequence relationships [82,91] and references therein) and the tree on the right represents host species relationships [81]. FIV polymerase sequences (158 amino acids included in analysis) were analyzed phylogenetically from nine feline species representing six out of the eight feline lineages [81]. Asterisks indicate significant bootstrap values (≥ 70%). The branch lengths are not in scale. Numbers next to node define estimated time of divergence for each the felid lineages and for the *Felidae*/*Hyaenidae *split in million years.

As already shown by several authors [74,91,93], the FIV phylogenies does not exactly mirror that of its feline host species (Figure 2). However, the relative differences in genetic diversity among FIV strains be interpreted in the context of the evolutionary and phylogeographical history of each host species. Indeed, in spite of that free-ranging individuals of many species harbor monophyletic, species-specific strain(s) of FIV, viruses isolated from different species seem to group more by geographic region of the host than in groupings concordant with the phylogenetic relationships of host species. Moreover, molecular analyses failed to resolve the origin domestic cat FIV strains as has been already shown by other studies [74,83]. The pattern of the strains infecting domesticated cat (FIV-Fca) which exhibit three monophyletic clades may due rapid viral diversification within the domestic cat world-wide due to the great number of individuals (some estimates put the domestic house cat population at 60 million and the feral cat population at the same number, that's 120 million animals) and to the trans-continental travels and traffics. The extreme divergence between the two highly FIV-Pco clades and the six FIV-Ple clades suggest an ancient origin of FIV infection of respectively, puma and lion [88]. Concerning FIV-Pco, this could be a consequence of two separate introductions of FIV within puma populations [83], whereas for African lion virus, each clades correspond with distinct geographic areas of endemicity [88]. The strains infecting cheetah (FIV-Aju) and leopard (FIV-Ppa) are closely related, in spite the fact that their hosts have evolved in distinctly different felid lineages, puma and cheetah are closely related, belonging to the puma linage, while lions and leopards are members of the Panthera lineage [81]. Moreover, cheetah and leopard could be sympatric; all these data suggest recent inter-species transmission. Due to the date of expansion of cheetah throughout Africa, the FIV-Aju emergence may have occurred within the last 10,000 years, perhaps acquired from leopards [93]. FIV-Oma is found in wild populations of the Eurasian Pallas cat [91], a species that arose during the late Pleistocene [81]. The monophyletic lineage of Pallas cat FIV-Oma and African lion FIV-Ple observed here could suggest more ancient inter-species transmission as the last time lions and Pallas cats were in geographic contact was during the Pleistocene when lion ranges spread throughout Asia, providing a possible opportunity for FIV transmission between these species [93]. In addition, FIV-Ccr occurs in spotted hyena, a species from the Hyaenidae family within carnivores that co-exist in the same habitats as most African felid species, affording opportunities for cross-species transmission. Interestingly, as already shown by Pecon Slattery et al. [93], all the FIV strains which infected Afro-Asian Feloidea constitute a monophyletic group. This grouping could suggest a common origin or/and old cross transmissions, in spite that in Asia, no wild seropositive individuals have been found in cheetahs, lions, leopards and hyenas which have a large Afro-Asian repartition [94]. Moreover, the geographic partitioning reflected in the amino acid phylogeny suggests evidence for an Old world/New world split (Figure 2 and [74,91]). Lastly, similarly to the cheetah/leopard case, two American species that evolved in distinctly different felid lineages (ocelot and jaguarondi), which have almost identical distribution, are infected with closely related viruses (FIV-Lpa and FIV-Hya, respectively) suggesting recent inter-species transmission. However, with few exceptions, the strong monophyletic origin of each species-specific strain suggests that FIV has rarely undergone effective transmission between species. In addition, the monophyly of FIV sequences within each species suggests that, in most cases, FIV has been successfully introduced once and adapted, expanded, and evolved within the host.

The precise origin of FIV emergence into Felidae is not easily discerned by viral phylogenetic analyses due to its recent and rapid evolution, and to cross-transmissions. According to Pecon-Slattery et al. [93], the widespread occurrence of FIV combined with large interspecies divergence in Africa would suggest that FIV arose in Africa rather than Asia. Moreover, an African origin of all lentiviruses may be posited, indeed, Simian lentiviruses are endemic in Africa infecting over 36 species of primates [95], and caprine arthritis-encephalitis virus (CAEV), bovine immune deficiency virus (BIV) and visna are present in Africa ungulate species [96] and references therein]). Moreover, the substantial genetic difference observed among FIV lineages in Africa is consistent with a long residence time within these species, and suggest global dissemination of FIV from Africa during felid transcontinental migrations into Eurasia and the Americas [81]. Moreover, the near absence of FIV in Asian species (except for the Mongolian Pallas cats) suggests that the virus did not originate along with ancestral felids of Asia which exclude that the FIV might have arisen in Asia along with the progenitor of modern felids 10.8 MYA. In addition, FIV related strains infect African feline species and the spotted hyena; however, FIV phylogenetic analyses do not support an ancient introduction of this virus to the Felidae and Hyaenidae (i.e., prior to the Felidae/Hyaenidae, using fossil, split is dated at about 47 million years ago) [97] but more probably, a recent African crossspecies transmissions. Lastly, the presence of FIV in both old and new world felids suggests that the current viruses may have descended from transmission events that occurred the last time felid species crossed the Bering Straits in the late Pleistocene (>12,000 years ago [81]), or earlier. By contrast, like the recent emergence of HIV in humans, domestic cat lentiviral infections are relatively new diseases, with more limited distribution and lower seroprevalence than infections noted in lions and pumas [74]. The domestic cat evolved as a unique felid lineage only around 10,000 year ago [45] from subspecies of wildcat *Felis silvestris *inhabiting Near East Asia [43]. Seroprevalence studies, suggest that FIV is present in nearly all of the close relatives of domestic cat (*Felis *genus [81]) including European wildcat *F. s. silvestris *[91,98,99]. However, concerning European wildcat, it is due to recent cross transmissions from feral or domestic infected cats. In Europe, hybridization between domestic cats and wildcats are well known [50,100,101], showing evidence that contacts between wild and domestic cats are not rare.

As FIV-infected wild felids are present in most of the world countries since at least the end of the last glaciation, it could be interesting to analyse the historical relationship between human and felids in relation with their serological status (Table 1). The exact history of human interaction with felids is still somewhat vague; however, as wild felid species are found in all parts of the world, except Greenland, Australia and Antarctica, suggesting that contacts between men and felids were probably very numerous during the last millennia. In spite that archaeological and historical records are sketchy, there are several evidence that throughout history people have had close relationships with felids. Moreover, given that, the single domestication event within the Felidae, apart from these modern hybrids, might suggest that this group is behaviourally poorly preadapted for domestication; it is all the more surprising that in a wide variety of cultures, over many centuries, particular felid species have been "tamed" as domestic pets. In addition, tamed felids have possibly lived in association with humans far earlier than archaeological and historical records imply. A comportemental study has evidenced that numerous species of small cats have an important preadaptation to domestication [102].

As summarized in the Table 1, in Afro-Asia, numerous felid species can be tamed including the four species with specific FIV. Cheetahs, which have been considered the easiest of the exotic cats to tame, have been tamed by several ancient Afro-Asian civilizations since 2500 to 5000 years ago [40,65,68,103,104]. Lions and leopards have been tamed since the beginning of Egyptian history (2800–2650 BC) [105,106]. Tigers were a popular animal in aristocratic collections in Asia for centuries [65],). Servals and caracals have been tamed in Egypt since at least at the 15^th ^century AD [106] and several centuries later, caracal have been trained for hunting in Asia [105,107-109]. The earliest remains of cats in domestic or tamed contexts from Egypt date from about 4000 to 3000 BC; moreover, archaeological remains of *F. chaus *and *F. s. lybica *have been found [52,110]. Pallas Cats (*F. manul*) have been reports of this cat being kept in a semi-domestic state in Central Asia [111]. More surprisingly, concerning an Afro-Asian non felid feliformia, there is evidence from paintings and bas-reliefs in tombs that in ancient Egypt striped hyenas were tamed and kept as pets, as well as being artificially fattened as food or for medical use [112,113].

In pre-Columbian times, relatively few animals were domesticated, and almost none of them extended beyond the geographic limits of their wild ancestors. However, jaguarondi and Geoffroy's cats have been partially domesticated as a rodents-catcher [114], and other American felids which are relatively easily tamed, like ocelot, margay, and puma have interacted with humans [65,115,116]. In summary, if except bobcats (however, young bobcats can be somewhat tamed), all the other American species bearing specific FIV have had closed relationships with natives [91,117].

This bibliographic analysis suggests that both in Afro-Asia and in America, numerous people could have been in contact with FIV. However, the principal criticism could be that most of the contacts with felids have restraints to wealthy people. If it is partially true for big cats as lion, leopard, puma and cheetah, but this is not the case for Pallas's cat, Geoffrey's cat and jaguarondi. In addition, four species (cheetah, leopard, lion and spotty hyena) with specific FIV were formerly widespread throughout western Asia and Africa. To date, none wild individuals of these species have been seropositive in Asia; however, at least four empires (Egyptian, Hyksosian, Achaemenian and Greek) have been on two continents, facilitating animal trade across the Sinai Peninsula and importation of African felids in Asian countries and vice-versa.

Moreover, concerning early European contacts with FIV-infected felids, the Romans displayed lions, tigers, leopards, cheetahs and other felids in menageries, pageants and arena combats [118], most of them having been caught in Africa and southwest Asia [53], but they were rarely tamed [106]. In the Roman Empire there were many amphitheatres, e.g. in the second century AD there were more than a hundred amphitheatres in Italy and a similar number in the rest of Europe [119]. In addition, there were similar numbers of circuses. The Romans systematically collected animals for display, entertainment and slaughter in arenas, theatres and amphitheatres throughout the Empire [120]. Even if the spectacles staged in Rome did not have an equivalent importance elsewhere in the Empire, in the arenas of this large city a great number of felids were massacred. For example, the dictator Sulla (93 B.C.) exhibited lions in the Rome's arena; in 55 B.C. under Pompey's reign on two occasions 500 and 410 leopards fought against Gaetulians armed with darts; in 46 B.C. Julius Caesar had 400 lions imported primarily from North Africa; and after Trajan's victory over the Dacians the games continued for 123 successive days when 11,000 animals were killed in the arena [120-124]. Caretakers could be bitten by these felids; moreover, captive felids could infect domestic cats and vice-versa, cross-species FIV transmission involving captive felids are well documented [74]. In addition, similarly to Simian retrovirus infections [125-128], human could be infected during hunting or cutting up, most of the felid species having always been very exploited for their pelts.

In summary, with exception of Oceania, historical regions with low or null frequency of *CCR5-Δ32 *allele coincide with historical range of the wild felid species which harbor species-specific FIVs (Figure 1B). Among these nine felid species, four of them have the largest distributions of the members of this family. Leopards have the largest distribution of any felid and were found from South Africa across that continent to the Middle East, Java, and northward to Siberia. According to historical records, lion populations have been distributed in Middle East to India and in Africa except in desert and rainforest habitats. The distribution of cheetahs was almost identical to that of lions, except that they have not been found in Europe, but that they were distributed in semi-deserts. Historically, pumas were found from the boreal forests of northern Canada to the tip of South America. Among the four other felid species, the Pallas' cats inhabited from the Caspian Sea area to parts of Western China through Southern Asia. In nearly half of its distribution range, they were sympatric with lions, cheetahs and/or leopards. The bobcat formerly ranged from southern Canada throughout most of the United States, south to central Mexico. The distribution of the ocelot was almost identical to that of jaguarondi; they were found from Arizona and south west Texas through Central America to South America except in high mountains or plateaux and in the extreme southern cone beyond approximately 45° latitude. In the past, lions and leopards lived in Balkans, but they were not numerous in the historical time and the last specimens became extinct about 2500–2000 years ago [129,130]. In Europe, only two species (Eurasian and Iberian lynx) and one subspecies (Eurasian wildcat) of wild felids live since historical times, and their seropositive level is null or very low and probably due to recent contamination by domestic cat [86,98,99].

## Discussion

Previous analyses suggested that in Europe the *CCR5-Δ32 *allele frequency is negatively correlated with colonization by ancient Mediterranean civilizations principally Romans [5]. We have the hypothesis that a zoonosis could have played a role in the decrease of the mutation frequency or in the absence of maintenance of the null allele if it would have appeared. As the cat spread throughout Europe is principally due to Romans, a cat zoonosis could be involved. Interestingly, to the exclusion of Oceania, in the countries in which FIV infected felids are found, the lower *CCR5-Δ32 *allele frequency is found in native human populations. Further bibliographic analyses are needed in order to know if FIV could infect human and also if the *CCR5-Δ32 *mutation can be unfavourable.

### Could FIVs infect humans?

More than half of the 1407 human pathogens are zoonotic [131] and recent epidemics such as HIV and severe acute respiratory syndrome (SARS) have changed the view we had about emerging infectious diseases; these epidemics showed evidences that animal reservoirs are important sources of new infectious threats to humans. Contacts between humans and animals are a crucial rate-limiting step in this process, although data describing the variables that influence animal-to-human transmission are relatively scarce. Therefore, a brief analysis of the data supporting cross-species transmissions of Simian retrovirus to humans can be instructive. Data on SIV/HIV dramatize this point; scientists now theorize that SIVs were transmitted from primates to humans on several occasions [132-138]. Although HIV causes AIDS in humans, SIV does not cause any disease in its natural hosts. However, it is not known exactly how HIV first entered the human population [139], eating raw monkey meat, drinking monkey blood, or perhaps through another method of direct exposure to monkey bodily fluids have been suggested as a possible source and remains the best candidate so far [125,134]. These hypotheses are supported by the fact that primate handlers and those who hunt and butcher "bushmeat" (the meat of wild animals that includes chimpanzees, gorilla and other monkeys) have detectable humoral and cell-mediated immunity to SIV. There are at least eight documented incidents of zoonotic transfer of SIV to humans [137] and two laboratory workers have been accidentally infected by SIV, one infection was cleared and the second (a human infection with SIVsmB670), caused a persistent asymptomatic infection [140-142].

In addition, the family of SIV is 1 out of 5 primate borne retroviruses known to infect humans. Simian (spumaretro-) foamy viral (SFV) infection, probably acquired through bites, has also been reported in 1 to 5% of persons occupationally exposed to non-human primates in zoos, primate centers and laboratories, mainly in Northern America but also in Europe (reviewed in [143]). Recently, naturally acquired SFV infections have been described in 1% of hunters living in Cameroon, Central Africa [125] and in one person with frequent contacts with *Macaca fascicularis *in a Indonesian temple [144]. In Cameroon, more than 60% of the population is directly exposed to fresh nonhuman primate blood and bodily fluids from hunting, butchering or petting [125,126]. Moreover, it has been recently demonstrated efficient transmission of SFVs to humans in natural settings in Central Africa, specifically following ape bites, and viral persistence in the human host [145]. There is currently no evidence of human-to-human transmission of SFV; however, only a few cases (*n *= 6) have had a short clinical follow-up [146-149]. Simian T-cell lymphotropic viruses (STLVs), enzootic in both Asian and African Old World monkeys and apes, may have repeatedly crossed the species barrier, the close relation between human and great ape primate T lymphotropic virus type 1 (PTLV-1) strains in Africa is suggestive of zoonosis [126,127], which might result from hunting and slaughter activities. In addition, serologic studies have demonstrated evidence of primate-to-human transmission of simian type D retrovirus (SRV), a retrovirus enzootic among Old World monkeys, in laboratory workers exposed to captive primates [150]. To date, no disease has been linked to human infection with this retrovirus.

To date, concerning the FIV, for which the host is phylogenetically more distant to human than monkeys, there is no evidence that it can infect or cause disease in humans. Researchers and veterinarians who have been bitten by FIV positive cats have been consistently tested negative for FIV [151]. However, FIV infection was assessed solely by serological tests, confirmation of direct exposure to the virus was limited, and prolonged periods between potential exposure and assessment of infection existed. FIV-specific antibodies were not detected in the cynomolgus macaques (*Macaca fascicularis*), in which FIV infection cause clinical signs, including depletion of CD4+ cells and weight loss, which are consistent with FIV infection; moreover, FIV genes expression has been found in necropsied tissues [63]. As the most obvious effects of FIV infection in macaques were observed early after exposure, the lack of serum detection suggests that seroconversion is not indicative of prior exposure to the virus. In addition, even if FIV is antigenically distinct from the primate lentiviruses, it shares many biological properties that manifest in its ability to infect productively both primary and immortalized primate cell lines *in vitro *[64,152-161]. In addition, a FIV strain which cannot naturally infect primate cells, when forced, preferred human cells to monkey cells [161] and the restricting effect of the host factor TRIM5α is fairly substantial in macaque cells, but is rather mild in human cells [162,163]. However, the ability of FIV to express its LTR in primate cells seems to vary depending upon the viral strain, the experimental protocol, and the cell line used. Most of the restriction to expression seems to be due to limitations imposed by promoter sequences residing within the U3 region of FIV LTR [158,159]. Once this restriction is overcome, FIV is able to express in a wide variety of cell types [64]. Moreover, it is likely that the determinants of feline cell tropism, such as envelope-mediated entry of target cells may also influence infection of primate cells by FIV, which must find cells that express the right combination of receptors and co-receptors [161]. While the chemokine receptor CXCR4 as an entry receptor and the tumor necrosis factor receptor CD134 have been well established as essential for FIVfca receptor-mediated cell entry, the receptor interactions of puma and lion FIVs are not identified, but in some cases appear to involve other cell surface determinants [73,164-170]. Moreover, a puma FIV isolate targeted gastrointestinal peripheral lymphoid tissues or other sites in a domestic cat infection model [171].

The use of CXCR4 and CD134 as receptors is compatible with our hypothesis, as well as, analogous to primate lentivirus receptor usage, the predominant FIVfca quasispecies changes during the course of FIV infection, in that isolates from terminally infected animals have been reported to be CD134 independent [168]. However, to date, there are no firm data to support a role for CCR5 in infections of feline cells [63,160,172], but a FIV strain could use human CCR5 to infect some human cells [161], nevertheless, this could be the result of a recent shift in coreceptor usage. In another hand, it has been reported that env deletion mutants of FIV have adapted to replicate in human cells [159]. Moreover, the increased cell death that preceded a loss of infectious FIV in infected human peripheral blood mononuclear cells supports previous findings that infection of human cells by FIV is cytopathic, which is probably due to the expression of FIV envelope glycoproteins [158]. FIV infection of relatively few cells in culture has been associated with increased cytotoxicity in feline cultures due to the release of cytotoxic molecules [173,174], which is similar to reports of other lentiviruses. Hence, it is conceivable that FIV-mediated cytotoxicity may limit the number of infected and potentially infectable cells leading to the loss of detectable FIV DNA in infected human cultures. So, even if there were not a true infection, a high rate of cellular death and/or an immunological depletion could be deleterious although the infection appeared to be clinically silent. Wolfe et al. [35] have delineated five stages in the transformation of an animal pathogen into a specialized pathogen of humans. According to these last authors, the present hypothesis of human infection by FIV would correspond to the stage 2: a pathogen of animals that, under natural conditions, has been transmitted from animals to humans ("primary infection") but has not been transmitted between humans ("secondary infection").

If the cause of the change of *CCR5-Δ32 *allele frequency was FIV infection, the characteristics of the virus that was present 2000–3000 years ago are unknown, especially since recombinations and cross-species transmissions have been shown for this virus. Discordant *env *phylogeny between *FIV*_*Ple *_subtypes reveals ancestral FIV recombination events in the wild [88]. It is probable, as with primate lentiviruses [74,175], that recombination plays a significant role in the evolution of FIV and that different evolutionary patterns would be seen within different viral regions. Although cross-species transmissions have been rare, they likely did occur in the past to produce a pattern of viral evolution in felids that does not completely match the evolution of the Felidae. One of the best examples is the position of hyena FIV-Ccr within felid FIV suggests increased opportunities for inter-species transmission due to a greater elapsed time since the virus entered and disseminated in African felids. Finally, there are now several examples of modern inter-species transmissions (Figure 1 and [82,91,176,177]). However, while there is one case of a free-ranging leopard cat that acquired FIVfca from a domestic cat [177], most cross-species transmissions of FIV have been documented in captive settings.

In natural settings there are substantial behavioural and ecological barriers to cross-species transmission of FIV, a pathogen requiring direct contact for infection to occur. The major mode of transmission for FIV in domestic cats is believed to be biting, although vertical transmission can also occur [178]. If we hypothesize that the FIV infected cats before their domestication, this suggests, after this last event, frequent transmissions of FIV by biting from cat to human. Even if it is speculative, several forms of infections could occur and it is important to underline that during Antiquity, the bodies of colonized people faced greater danger from infections new to their immune systems and that numerous infectious diseases have profoundly affected human populations. Infections could induce fever, this might pass unnoticed and moreover, several prolonged or not fevers occurred relatively frequently during Antiquity [179] and still today, fevers of unknown origin are numerous and several of them are probably zoonoses [131]. Moreover, even if the virus cannot infect productively human cells, it could induce cell death. The *in vitro *lytic properties of this virus in monkey and human cells suggest possible biological abnormalities associated with human FIV infection. Moreover, infections usually benign alone could have more severe effects on people which were co-infected by several epidemic or endemic pathogenous agents.

Moreover, cat zoonoses can be transmitted to man [41,42] and the hypothesis of the role of FIV remains putative. However, the implication of a feline retrovirus could be plausible; indeed, three other species of feline retroviruses, feline foamy virus (FeFV), feline sarcoma virus (FeSV) and Feline leukemia virus (FeLV) can replicate in some human cell cultures with generally production of infectious virus and could sometimes produce morphological cell change [151,180-192]. Moreover, cat horizontal transmission of FeLV by cat fleas has also been demonstrated [193] and FeSV can also induce malignant tumours in non felid mammalian including monkeys [194]. To date, there has been no evidence of infection of feline retrovirus in humans so far. However, all these reports suggest that numerous cat pathogen agents could have played a role in the putative decrease of the null allele frequency.

### Could the null allele be unfavourable?

As other receptors for inflammatory chemokines, CCR5 contribute to leukocyte recruitment in a number of inflammatory diseases (reviewed in [195]). However, owing to the redundancy of the chemokine system, CCR5 could only play a modest role, and blocking CCR5 was predicted to be safe because individuals lacking CCR5 develop normally and seem healthy. Nevertheless, over the years, the CCR5-Δ32 allele has been linked, using epidemiologic studies, with several non infectious human diseases, including multiple sclerosis and schizophrenia [196-198] but the associations have generally been weak or inconsistent between these studies. In another hand, in mouse models of infection, CCR5 has been implicated in host defense against Influenza A virus, *Listeria, Trypanosoma cruzi*, *Toxoplasma gondii*, *Cryptococcus neoformans *and *Chlamydia trachomatis *[199-205]. In humans, similarly to HIV-1, *CCR5-Δ32 *carriers also have a decreased likelihood of contracting hepatitis B virus [206], but these carriers improved outcomes during hepatitis C virus infection [207] and tick-borne encephalitis virus infections (TBEV) [208]. Moreover, it has been reported that *CCR5-Δ32 *homozygosis was strongly associated with symptomatic West Nile virus (WNV) infection [28,209], consistent with a previous finding that CCR5 was a crucial antiviral and survival factor in WNV infection in mice [210]. WNV and TBEV are members of the same family (Flaviviridae) and share certain similarities between them. Interestingly, like most of the infectious agents, flavivirus and influenza viruses are endemic in several tropical and subtropical regions and probably CCR5 is implicated in the defense against several other tropical viruses; this could perhaps explain why the *CCR5-Δ32 *allele frequency is relatively weak in these areas, even if this or another null mutation has arisen, they could be rapidly under selected. If our hypothesis is correct, this could explain the quasi-null allele frequency in Australia [211], in spite that the Aborigines have not been in contact with felids during approximately 50,000 years [212]. In the context of infectious diseases, CCR5 comprises positive and negative elements that ultimately contribute to the evolution of the gene over time. In flavivirus infections and putatively ancient cat zoonosis pathogenesis, CCR5 is antimicrobial, whereas in HIV pathogenesis, CCR5 is promicrobial.

### Can archaeologists excavate evidence of cats' role in the human CCR5-Δ32 allele frequency?

Future studies on ancient DNA will confirm or reject our hypothesis which include a great *CCR5-Δ32 *allele frequency in the ancient European population, followed by a progressively decrease of the frequency southwards due indirectly to Romans and other colonizers which have helped spread a possible cat zoonosis to native populations. These future analyses could also give data for characterisation of ancient European pathocenosis compositions including the genetic responses and changes to epidemic and endemic diseases. Indeed, whereas evolutionary information derived from present-day DNA sequences is, by necessity, indirect, ancient DNA sequences provide a direct view of past genetic variants and infectious agents. Moreover, technical advances in DNA extraction, multiplex DNA amplification and high-throughput sequencing have recently opened new horizons in ancient genomics (references there in [213]), and studies to elucidate the genetic basis of the environmental adaptations of the human ancestors, compared to humans today is now possible. The presence and frequencies of the *CCR5-Δ32 *variant in past human populations has been studied by several authors. The results of these studies have argue against the possibility that plague was a major selective force that caused a rapid increase in *CCR5-Δ32 *gene frequencies within European populations [22,214] and have pushed the dating of the *CCR5-Δ32 *allele back to around 5000 BC [11].

Moreover, sequencing of complete genome of *Homo sapiens neanderthalensis *is underway [215,216] and could give interesting data concerning the origin of the null allele. Indeed, as Neanderthals are the extinct hominid species most closely related to contemporary humans, the continuation of the Neanderthal genome project provides a unique opportunity to identify genetic changes that are specific to modern humans [215]. Dating such genomic events would help to interpret these changes mechanistically. In addition to the different methods of age estimation based on allele frequencies and sequence comparison between species, conclusive data from the analysis of prehistoric remains of members of the genus *Homo *(e.g. from humans and Neanderthals) would help to date such events by determining the presence and frequency of genomic variants. Moreover, Currat and Excoffier [217] using a method, which assumed environmental homogeneity, have simulated the range expansion of modern humans into Europe under realistic demographic scenarios to investigate potential admixture between colonizing humans and resident Neanderthals. Their simulations indicated that even with only a few admixture events, the contribution of Neanderthal genes to the current human gene pool should be large because new genes (which have a Neanderthal origin) have a high probability of persistence when entering a progressively expanding (modern human) population compared with those entering a stationary population. In a recent review, Hodgson and Disotell [218] have concluded that "it seems unlikely that Neanderthals contributed any substantial fraction of modern variation and it remains to be seen whether any adaptive alleles crossed the human-Neanderthal species boundary". Moreover, more recent major events in human evolution, such as the re-colonization of northern latitudes after the Ice Ages, could also be taken into account.

In addition, the analysis of the DNA of ancient micro-organisms in archaeological and palaeontological human and animals remains can contribute to the understanding of issues as different as the spreading of a new disease. The molecular resolution of extinct species' genomes raises the hope of discovering infectious agents and pathogens that might have played a regulatory role in historic ecosystems. Potentials, and sometimes pitfalls, of this research field are illustrated by the results of the various research works performed on ancient DNA. For example, DNA of bacteria of the genus *Bartonella *responsible of chronic bacteremia and which have mammalian reservoirs including cats has been detected in a human and a cat who lived respectively 4000 and 800 years ago [219,220]. Moreover, the finding of ancient human T cell leukemia virus type I (HTLV-1) long terminal repeat (LTR) DNA sequences in association with a 1500-year-old Chilean mummy [221,222], even if it has stirred vigorous debate shows that ancient provirus sequences will become available in the future. Cumulative research on felid natural history, evolution, phylogeography and ancient DNA analyses will provide important context for FIV emergence. Ancient DNAs from felids are useful not only to phylogenetic analysis but also to population genetic approaches that may increase our understanding of the incipient extinction of modern species [71,223,224]. Moreover, the potential role of extinct felids, such as the saber-tooth species, which co-existed with modern felids until around the end of the Pleistocene [69] in FIV origin and its dissemination could be known.

## Conclusion

In this study, we have proposed the hypothesis that in Europe, the actual European CCR5 allelic frequencies are the result of a negative selection due to a disease spreading (ostensibly by the Roman Empire or some other colonizers). A cat zoonosis could be the most plausible hypothesis and even if it is speculative, the implication of FIV added to possible deleterious effects of the null allele mutation has been suggested. Future studies will prove or dismiss if in FIV pathogenesis, CCR5 can play an antimicrobial role. Moreover, this study shows that in the future all pieces of the puzzle could be put together to see the whole picture of the *CCR5-Δ32 *allele evolution.

Bibliographical analysis shows evidence that species-specificity of FIV might be less stringent than previously considered. The abundance of studies demonstrating the capacity of viruses, including retrovirus, to cross species raises questions about ongoing transmissions and renders the study of the adaptations required for viruses to be transmitted from one host species to another increasingly relevant. In addition, although bibliographical analysis shows that the FIV has the ability to infect primate cells *in vivo*, it is not our intent to suggest that FIV represents a health hazard. However, the apparent lack of pathogenicity of FIV infection in humans, which is still based on a limited number of cases, contrasts strongly with the *in vitro *lytic properties of these viruses in primate cells. Moreover, as the analyses concern only healthy persons this induces an important bias. Although the risks for human are considered extremely small, from a public health perspective it is often recommended that immunosuppressed people should have limited contact with infected cats. FIV infection in immunocompromised persons, especially those with HIV infection, could also heighten public health concerns because such coinfection is probable during cohabitation with infected pets.

In addition, scientific evidence for the ancient spread of a resistance allele or a pathogenic agent could become available through research on ancient DNA and this research field could be determinant in the comprehension of the interrelations with human genome, pathogenic agents and their hosts in the last millennia. Recent advances in ancient-DNA extraction have made it possible to retrieve substantial amounts of ancient DNA sequences from at least Pleistocene remains in order to analyse the pathocenoses and the corresponding genetic responses. As most of the human diseases are zoonoses, analyses of human and animal remains must be made in conjunction.

This study shows also evidence that only an integrated multidisciplinary approach has enabled us to understand the evolutionary history of the *CCR5-Δ32 *allele.

## Methods

### Data sources

We have compiled bibliographical data concerning the past distribution of felids which are now infected by species-specific pathogenic agents. Species descriptions and all references are in Table 1.

### Sequence analyses

All the FIV Pol protein sequences have been extracted from GenBank. These sequences have been aligned with the BioEdit software [225]. Phylogenetic analyses were performed using the Neighbor-Joining (NJ) method [226] in PHYLIP version 3.6 alpha 3 [227] accessed at . Robustness of nodes was estimated by running a bootstrap test with 100 replicates.

## Competing interests

The author declares that he has no competing interests.
